# 5-Hydroxymethylfurfural Alleviates Inflammatory Lung Injury by Inhibiting Endoplasmic Reticulum Stress and NLRP3 Inflammasome Activation

**DOI:** 10.3389/fcell.2021.782427

**Published:** 2021-12-13

**Authors:** Hang Zhang, Zheyi Jiang, Chuanbin Shen, Han Zou, Zhiping Zhang, Kaitao Wang, Renren Bai, Yanhua Kang, Xiang-Yang Ye, Tian Xie

**Affiliations:** ^1^ School of Basic Medical Science, Hangzhou Normal University, Hangzhou, China; ^2^ School of Pharmacy, Hangzhou Normal University, Hangzhou, China; ^3^ Key Laboratory of Elemene Class Anti-cancer Chinese Medicine of Zhejiang Province, Hangzhou Normal University, Hangzhou, China; ^4^ Engineering Laboratory of Development and Application of Traditional Chinese Medicine from Zhejiang Province, Hangzhou Normal University, Hangzhou, China; ^5^ Department of Cardiology, Shanghai Ninth People's Hospital, Shanghai Jiaotong University School of Medicine, Shanghai, China; ^6^ Collaborative Innovation Center of Traditional Chinese Medicines from Zhejiang Province, Hangzhou Normal University, Hangzhou, China

**Keywords:** 5-hydroxymethylfurfural, acute lung injury, NLRP3 inflammasome, endoplasmic reticulum stress, inflammation

## Abstract

5-Hydroxymethylfurfural (5-HMF) is a common reaction product during heat processing and the preparation of many types of foods and Traditional Chinese Medicine formulations. The aim of this study was to evaluate the protective effect of 5-HMF on endotoxin-induced acute lung injury (ALI) and the underlying mechanisms. Our findings indicate that 5-HMF attenuated lipopolysaccharide (LPS)-induced ALI in mice by mitigating alveolar destruction, neutrophil infiltration and the release of inflammatory cytokines. Furthermore, the activation of macrophages and human monocytes in response to LPS was remarkably suppressed by 5-HMF *in vitro* through inhibiting the NF-κB signaling pathway, NLRP3 inflammasome activation and endoplasmic reticulum (ER) stress. The inhibitory effect of 5-HMF on NLRP3 inflammasome was reversed by overexpressing ATF4 or CHOP, indicating the involvement of ER stress in the negative regulation of 5-HMF on NLRP3 inflammasome-mediated inflammation. Consistent with this, the ameliorative effect of 5-HMF on *in vivo* pulmonary dysfunction were reversed by the ER stress inducer tunicamycin. In conclusion, our findings elucidate the anti-inflammatory and protective efficacy of 5-HMF in LPS-induced acute lung injury, and also demonstrate the key mechanism of its action against NLRP3 inflammasome-related inflammatory disorders via the inhibition of ER stress.

## Introduction

Acute lung injury (ALI) is a debilitating disease characterized by severe pulmonary lung inflammation and air leak in the upper lobe, which typically lead to hypoxemic respiratory failure in critically ill patients (Moazed and Calfee, 2014). Multiple endogenous inflammatory mediators and exogenous agents can trigger inflammatory response and vascular leak in the lungs (Wu et al., 2018), including pneumonia and pulmonary contusion sepsis, inhalation of noxious gases, and reperfusion (Moazed and Calfee, 2014). Lipopolysaccharide (LPS, also known as endotoxin) is the major toxic component present in the outer membrane of Gram-negative bacteria that causes considerable tissue injury and accelerates ALI pathogenesis (Xu et al., 2014; Kim et al., 2015a). It is a typical macrophage activator that stimulates the innate immune system through multiple downstream signaling pathways (Xu et al., 2014). Given the lack of effective drugs against ALI, it is crucial to develop novel therapeutic strategies and explore the molecular pharmacological mechanisms.

LPS-induced ALI is mediated by proinflammatory cytokines and mediators such as nitric oxide (NO), interleukin-6 (IL-6), tumor necrosis factor-α (TNF-α), IL-1β and IL-8, which are produced by the alveolar macrophages (Wu et al., 2018) following activation of the mitogen activated protein kinases (MAPK) and NF-κB signaling pathways (Xu et al., 2014; Kim et al., 2015a). LPS is recognized by toll-like receptor 4 (TLR4) that is expressed on the cell membrane of various immune cells (Lu et al., 2008), and the ensuing stimulation of TLR4 activates the NF-κB and MAPKs (ERK, JNK and p38 kinases) signaling pathways. Therefore, inhibiting these pathways may alleviate LPS-induced inflammation and pulmonary dysfunction.

Studies show that the nucleotide-binding domain (NOD)-like receptor protein 3 (NLRP3) inflammasome plays a pivotal role in the pathogenesis of ALI. The NLRP3 inflammasome is a protein complex consisting of NLRP3, pro-caspase-1 and apoptosis-associated speck-like protein containing a C-terminal caspase recruitment domain (ASC). ASC promotes activation of the NLRP3 inflammasome (Lamkanfi et al., 2010) by mediating caspase-1 maturation. The activated caspase-1 cleaves pro-IL-1β and pro-IL-18 to their respective mature forms, eventually triggering cytokine secretion and inflammatory responses (Compan et al., 2015). Since inactivation of NLRP3 inflammasome has been shown to protect against hyperoxia-induced ALI (Fukumoto et al., 2013), NLRP3 inflammasome may be a viable target for developing potential candidate drugs to treat ALI.

Endoplasmic reticulum (ER) stress is a process wherein misfolded or unfolded proteins accumulate in the ER and trigger the unfolded protein response (UPR). In mammalian cells, ER stress involves three signaling cascades mediated by pancreatic endoplasmic reticulum kinase (PERK), inositol-requiring enzyme 1 (IRE1) and activating transcription factor 6 (ATF6), which is activated by the ER chaperone glucose regulated protein 78 (GRP78). These UPR sensors regulate multiple downstream components, such as the alternative mRNA splicing of X-box binding protein 1 (XBP1), and the expression of eukaryotic translation initiation factor 2 subunit alpha (eIF2α) and C/EBP-homologous protein (CHOP), which upregulate several target genes to restore ER homeostasis (Zhang and Kaufman, 2008). There is evidence indicating that ER stress is involved in LPS-induced lung inflammation and ALI. For instance, the ER stress components GRP78 and CHOP are upregulated in an LPS-induced ALI rodent model (Kim et al., 2013). In addition, stimulation of the alveolar epithelial cell line A549 with LPS enhanced PERK and elF2α phosphorylation, and the nuclear translocation of ATF4 (Ye et al., 2015). Studies increasingly show that ER stress is associated with the activation of NLRP3 inflammasome, and its suppression can inactivate the NLRP3 inflammasome (Oslowski et al., 2012). In addition, inhibition of ER stress can attenuate LPS-induced ALI *in vivo* and *in vitro* (Kim et al., 2015b; Zeng et al., 2017). Taken together, the ER stress pathway is a promising therapeutic target in NLRP3 inflammasome-related inflammatory diseases.

**FIGURE 1:**
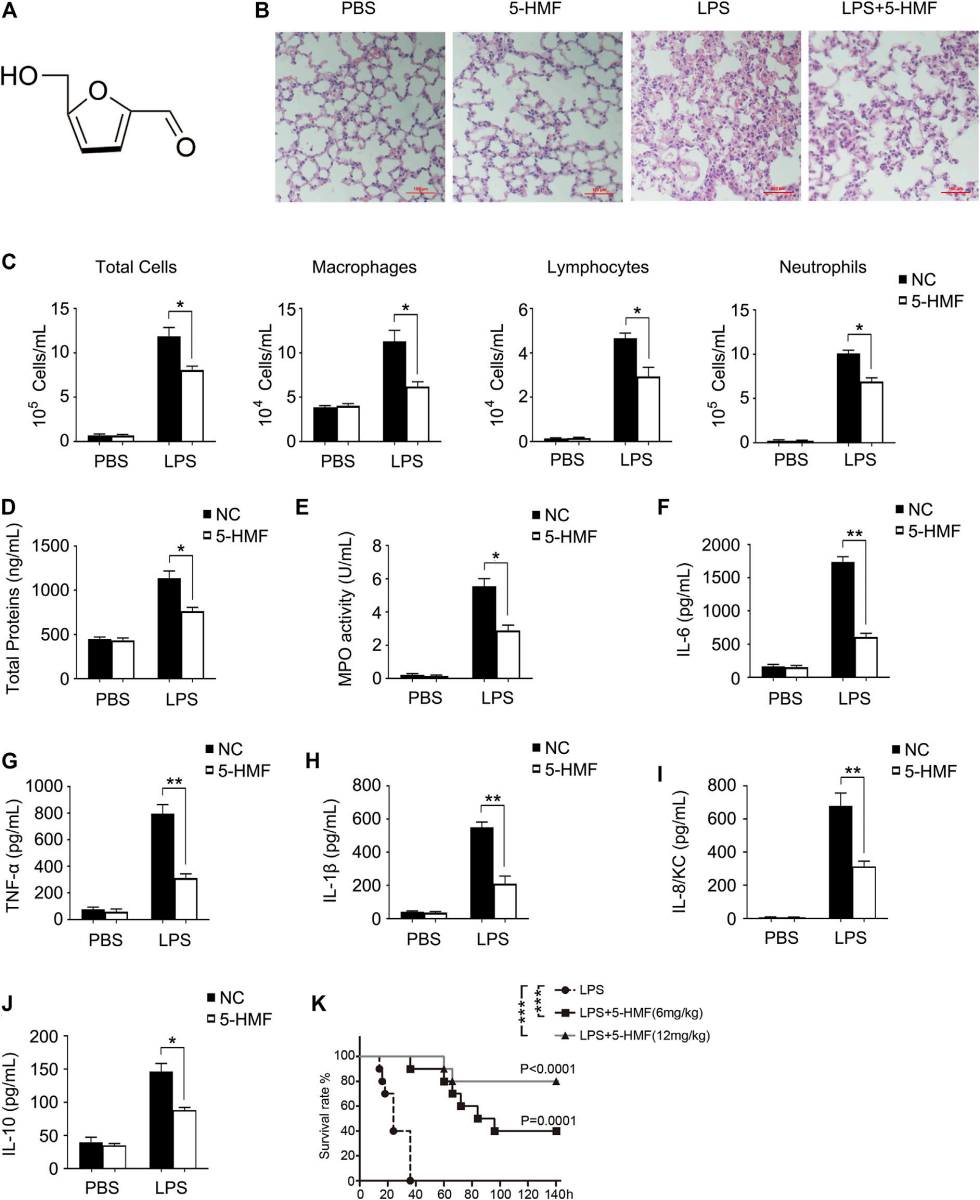
5-HMF attenuates organ damage, serum cytokine secretion and mortality in LPS-challenged mice. **(A)** Chemical structure of 5-HMF. **(B–J)** C57BL/6J mice (5 mice/group) pretreated with 5-HMF (12 mg/kg, i. p.) or PBS were injected intraperitoneally with LPS (1 mg/kg) or PBS. **(B)** Representative images of H&E stained lung tissue sections from the indicated groups (×200), Scale bar, 100 μm. **(C)** Cellular changes in BALF. **(D)** Protein content in BALF. **(E)** MPO activity of lung tissues. **F–J)** Serum levels of IL-6 **(F)**, TNF-α **(G)**, IL-1β **(H)**, IL-8/KC **(I)** and IL-10 **(J)**. All results are expressed as the mean ± SD: * and ** indicate *p* < 0.05 and *p* < 0.01 respectively by Student’s t-test. **(K)** Kaplan−Meier survival plots of C57BL/6J mice (10 mice/group) injected intraperitoneally with LPS (25 mg/kg). Survival status was recorded for 6 days and data were analyzed using the Log-Rank test (*n* = 10 mice per group; **p < 0.01).

Common heterocyclic Maillard reaction of sugars at high temperatures produces 5-hydroxymethylfurfural (5-HMF, Figure 1A), a furan-containing aldehyde which is present in various sacchariferous foods such as honey, coffee, fruit juices, dried fruits and baked foods that typically undergo thermal processing and long-term storage (Anese et al., 2013; Zirbes et al., 2013). 5-HMF has also been detected in various heat-processed Traditional Chinese Medicine (TCM) formulations including black garlic extracts (Kim et al., 2011) and *Codonopsis pilosula* (Feng et al., 2017). Studies have reported antioxidant (Zhao et al., 2013), anti-proliferative (Zhao et al., 2013) and cardioprotective effects (Wölkart et al., 2017) of 5-HMF. In addition, 5-HMF protects against alcoholic liver oxidative injury (Li et al., 2015), and exerts an anti-inflammatory effect on human umbilical vein endothelial cells (Kim et al., 2011) and LPS-stimulated RAW 264.7 macrophages (Kong et al., 2019). However, no study so far has reported a therapeutic role of 5-HMF in ALI. In this study, we analyzed the protective effect of 5-HMF against lung inflammation and injury, and found that 5-HMF inhibited the TLR-driven inflammatory response in macrophages by inactivating the NF-κB signaling pathway. Moreover, 5-HMF treatment significantly inhibited NLRP3 inflammasome activation in LPS-primed macrophages in response to ATP or nigericin stimulation. ER stress was identified as the central signaling cascade regulated by 5-HMF in LPS-stimulated macrophages, which was essential for its inhibitory effect on NLRP3 inflammasome activation. Our data suggests that 5-HMF suppresses the LPS-induced inflammatory response by blocking the NF-κB/NLRP3 inflammasome cascade through attenuation of ER stress. These findings provide insights into the mechanisms underlying the potential therapeutic effect of 5-HMF in ALI and related diseases.

## Materials and Methods

### Reagents and Antibodies

5-HMF (purity >99.5%), LPS (055:B5), Griess reagent, 4’, 6-diamidino-2-phenylindole (DAPI) and Fluo-3 AM were purchased from Sigma-Aldrich Co. (St. Louis, MO, United States). The anti-β-actin antibody (A5441) was purchased from Sigma Aldrich (St. Louis, MO, United States). Antibodies against NLRP3 (#15101), mouse IL-1β (#12242), human IL-1β (#12703), mouse ASC (#67824), human ASC (#13833), GPR78 (#3177), ATF4 (#11815), CHOP (#2895, for western blotting), eIF2α (#5324), phosphorylated eIFα (#3398) and PERK (#3192) were purchased from Cell Signaling Technology (Danvers, MA, United States). Antibodies against phosphorylated PERK (PA5-40294) and CHOP (MA5-32571, for immunohistochemistry) were purchased from Invitrogen (Carlsbad, CA, United States), and those specific for caspase-1 (ab179515), IRE1α (ab37073) and phosphorylated IRE1α (ab48187) were purchased from Abcam (Cambridge, UK). DyLight488-conjugated anti-rabbit IgG, horseradish peroxidase (HRP)-conjugated goat anti-mouse IgG and HRP-conjugated goat anti-rabbit IgG antibodies were purchased from MultiSciences Biotech (Hangzhou, China).

### Animals and Experimental Protocol

C57BL/6J mice (6–8 weeks old, weighing 20 ± 3 g, male) were purchased from SLAC Laboratory Experimental Animal Co. (Shanghai, China) and housed at the Animal Center of Hangzhou Normal University. All animal experiments were approved by the ethics committee for Animal Care and the Use of Laboratory Animals, Hangzhou Normal University. To assess the effect of 5-HMF on LPS-induced septic shock, the mice were randomly divided into the PBS control, 5-HMF, LPS and LPS+5-HMF groups (*n* = 5 each). The mice were injected intraperitoneally with 200 μL PBS or 100 μL 12 mg/kg 5-HMF as appropriate, followed by 100 μL PBS or LPS (1 mg/kg) i. p. after 1 h. The mice were sacrificed 16 h after the LPS challenge, and blood sera, broncho-alveolar lavage fluid (BALF) and lung tissues were harvested for further analysis of cytokine production, cell infiltration and MPO activity in BALF, and histological staining. The cytokines level in sera and the MPO activity were measured by ELISA. For cell differentials examination, total cell numbers were counted with a Nucleocounter (Thermo Fisher). Smears of BALF cells were prepared by cytospin, and stained with Diff-Quik solution (Saint-bio co., Shanghai, China) to examine cell differentials. To determine the role of ER stress in the above model, tunicamycin (TM)-treated groups were also included. The mice were randomly divided into the PBS control, LPS, LPS+5-HMF, LPS+5-HMF + TM and LPS + TM (*n* = 5 each). The mice were injected with PBS/5-HMF as described, and 0.2 mg/kg TM was injected i. p. 1 h before LPS induction. The mice were sacrificed 16 h after the LPS challenge, and samples were collected for the followed examinations as above.

For survival analysis, the mice were injected with 5-HMF (6 or 12 mg/kg, i. p.) or PBS, followed by LPS (25 mg/kg), and the survival statuses of each group was recorded at different time points as described previously (Kang et al., 2018).

### Cell Culture

The BMDMs were isolated from C57BL/6J mice as previously described (Kang et al., 2018). Briefly, the bone marrow was flushed out from the femurs and centrifuged at 1,000 rpm for 5 min. The harvested cells were resuspended and cultured in Dulbecco’s Modified Eagle Medium (DMEM, Life Technologies) supplemented with 10% (v/v) heat-inactivated FBS and 20% conditioned medium of L929 cells. The cells were harvested 6 days later for subsequent experiments. The THP-1 and RAW264.7 cell lines were obtained from the American Type Culture Collection (ATCC, Manassas, VA). THP-1 cells were cultured in RPMI-1640 supplemented with 10% heat-inactivated FBS and 50 μM 2-mercaptoethanol, and differentiated into macrophages by incubating with 10 ng/ml PMA for 16 h. RAW264.7 was cultured in DMEM with 10% (v/v) heat-inactivated FBS. All cells were maintained at 37°C in a humidified incubator under 5% CO_2_.

### Cell Viability Assay

The viability of the suitably treated cells was determined using the CellTiter 96 Aqueous Non-Radioactive Cell Proliferation Assay kit (MTS; Promega, Madison, WI, United States) (Lou et al., 2021). The cells were seeded into 96-well microplates and treated with 0, 5, 10, 25, 50 and 100 μg/ml 5-HMF for 48 h. After adding 20 µL MTS reagent per well, the cells were further incubated for 2 h. The absorbance of each well was measured at 490 nm and the inhibition rate was calculated from three independent experiments.

### Ribonucleic Acid Isolation, Real Time-Polymerase Chain Reaction and Quantitative Real Time Polymerase Chain Reaction

RNA was isolated from the cells using TRIzol reagent (Invitrogen, Carlsbad, CA) according to the manufacturer’s instructions. Complementary DNA (cDNA) was synthesized from 0.5 μg total RNA by reverse transcriptase (Takara, Dalian, China). SYBR Green PCR Master Mix (Bio-Rad) was used for quantitative real tiem PCR, and relative gene expression levels were determined with the ΔΔCt method using GAPDH as the endogenous control. The following primers were used: mouse IL-6 forward 5′-CTG​CAA​GAG​ACT​TCC​ATC​CAG-3′ and reverse 5′-AGT​GCA​TCA​TCG​TTG​TTC​ATA​C-3’; human IL-6 forward 5′- ACT​CAC​CTC​TTC​AGA​ACG​AAT​TG-3′ and reverse 5′-CCA​TCT​TTG​GAA​GGT​TCA​GGT​TG-3’; mouse TNF-α forward 5′-AAG​GCC​GGG​GTG​TCC​TGG​AG-3′ and reverse 5′-AGG​CCA​GGT​GGG​GAC​AGC​TC-3’; human TNF-α forward 5′-GAG​GCC​AAG​CCC​TGG​TAT​G-3′ and reverse 5′-CGG​GCC​GAT​TGA​TCT​CAG​C-3’; mouse IL-1β forward 5′-AAC​CTC​ACC​TAC​AGG​GCG​GAC​TTC​A-3′ and reverse 5′-TGT​AAT​GAA​AGA​CGG​CAC​ACC-3’; human IL-1β forward 5′-ATG​ATG​GCT​TAT​TAC​AGT​GGC​AA-3′ and reverse 5′-GTC​GGA​GAT​TCG​TAG​CTG​GA-3’; mouse inducible nitric oxide synthase (iNOS) forward 5′-GTT​CTC​AGC​CCA​ACA​ATA​CAA​GA-3′ and reverse 5′-GTG​GAC​GGG​TCG​ATG​TCA​C-3′ human iNOS forward 5′-AGG​GAC​AAG​CCT​ACC​CCT​C-3′ and reverse 5′-CTC​ATC​TCC​CGT​CAG​TTG​GT-3′ mouse IL-10 forward 5′-CTT​ACT​GAC​TGG​CAT​GAG​GAT​CA-3′ and reverse 5′-GCA​GCT​CTA​GGA​GCA​TGT​GG-3’; human IL-10 forward 5′-CTT​ACT​GAC​TGG​CAT​GAG​GAT​CA-3′ and reverse 5′-TCA​CAT​GCG​CCT​TGA​TGT​CTG-3’;mouse C-X-C motif chemokine ligand 1 (Cxcl1) forward 5′-TGA​GAG​TGA​TTG​AGA​GTG​GAC-3′ and reverse 5′-AAC​CCT​CTG​CAC​CCA​GTT​TTC-3’; mouse CHOP forward 5′-AAG​CCT​GGT​ATG​AGG​ATC​TGC-3′ and reverse 5′-TTC​CTG​GGG​ATG​AGA​TAT​AGG​TG-3’; mouse GPR78 forward 5′-ACT​TGG​GGA​CCA​CCT​ATT​CCT-3′ and reverse 5′-GTT​GCC​CTG​ATC​GTT​GGC​TA-3’; mouse GAPDH forward 5′- AGG​TCG​GTG​TGA​ACG​GAT​TTG-3′ and reverse 5′-TGT​AGA​CCA​TGT​AGT​TGA​GGT​CA-3’; human GAPDH forward 5′-GGA​GTC​AAC​GGA​TTT​GGT-3′ and reverse 5′-GTG​ATG​GGA​TTT​CCA​TTG​AT-3’.

To evaluate relative expression levels of XBP1u/XBP1s, RT-PCR analysis was performed using Primerstar PCR Mix (Takara) as described previously (Mimura et al., 2012). Murine XBP1 primer sequences were as follows: forward 5′-ACA​CGC​TTG​GGA​ATG​GAC​AC-3′ and reverse 5′-CCA​TGG​GAA​GAT​GTT​CTG​GG-3’. GAPDH was used as a loading control and PCR products were analyzed on a 3.5% agarose gel.

### Detection of Myeloperoxidase Levels, Enzyme-Linked Immunosorbent Assay and Western Blotting

Lung myeloperoxidase (MPO) levels were determined using mouse MPO ELISA kit (Hycult Biotech) according to the manufacturer’s instructions (Yu et al., 2014). The levels of different cytokines in the culture supernatants and sera were measured using specific ELISA kits according to the manufacturer’s instructions. ELISA kits for murine and human IL-6 and TNF-α were purchased from Invitrogen (Carlsbad, CA), ELISA kits for murine and human IL-1β were purchased from R&D Systems (Minneapolis, MN), ELISA kits for murine and human IL-10, murine KC/IL-8 and human IL-10 were purchased from Neobioscience (Shenzhen, China). For western blotting, equivalent amounts of protein were separated by SDS-PAGE, transferred to polyvinylidene fluoride membranes, and incubated with specific antibodies to analyze the specific protein levels as described previously (Dong et al., 2020).

### Over Expression of ATF4 and CHOP in BMDMs

Murine ATF4 and CHOP mRNAs were transfected into the BMDMs following the procedure described by Herb et al. (2019). The total mRNA of the BMDMs were reverse transcribed into cDNAs using a Reverse Transcription kit (Takara), and the ORF sequences of ATF4 and CHOP were amplified by PCR using the following primers: ATF4: forward: 5′-TCA​GAA​TTC​ATG​ACC​GAG​ATG​AGC​TTC​CT-3′ and reverse 5′-TAT​CTC​GAG​TTA​CGG​AAC​TCT​CTT​CTT​CC-3’; CHOP: forward: 5′-TCA​GAA​TTC​ATG​GCA​GCT​GAG​TCC​CTG​CCT​TT-3’; and reverse 5′-TAT​CTC​GAG​TCA​TGC​TTG​GTG​CAG​GCT​GAC-3’. The respective ORFs were cloned into the pcDNA3.1 vector downstream of the T7 promoter. The plasmids were then linearized by XhoI digestion, and were used as a template to generate the fragment DNA containing the T7 promoter and the ORFs of ATF4 or CHOP by PCR amplification, the following primers specific for the flanking sequences were used: forward: 5′-AAA​TTA​ATA​CGA​CTC​ACT​ATA​GGG​AG-3’; reverse: 5′-GCT​GAT​CAG​CGG​GTT​TAA​ACG​G-3’. The PCR product was purified and transcribed *in vitro* using the HiScribe T7 *in vitro* mRNA transcription kit (New England BioLabs). The mRNA was purified using MEGAclear transcription clean-up kit (ThermoFisher Scientific) and transfected into BMDMs at the dose of 200 ng/10^5^ cells using the jetMESSENGER mRNA transfection buffer and reagent (Polyplus transfection) according to manufacturers’ instructions.

### Luciferase Reporter Gene Assay

To determine NF-κB reporter activity, the pGL3-NF-κB reporter plasmid and the pRL-TK-Renilla-luciferase plasmid were co-transfected into RAW264.7 cells using X-tremeGENE DNA transfection reagent (Roche). After 24 h, the cells were treated with PBS or different concentrations of 5-HMF (0, 10, 25 and 50 μg/ml), followed by stimulation with LPS (100 ng/ml) for 6 h. The cells were harvested and lysed for dual luciferase assays (Promega) according to the manufacturer’s instructions.

### Immunofluorescence Staining

ASC-speck formation was detected by immunofluorescence staining. Briefly, 10-mm cell culture slides were placed at the bottom of 6-well cell culture plates, and BMDMs or PMA-primed THP-1 cells were seeded at the density of 5×10^6^/ml per well. After the suitable treatments, the cells were fixed overnight in 4% paraformaldehyde, and incubated sequentially with anti-mouse or anti-human ASC antibody (1:100), followed by staining with AlexaFluor488-conjugated goat-anti-mouse IgG antibody (1:1,000). The nuclei were counterstained with DAPI (2.5 μg/ml). The ASC speck formation were observed and counted under a fluorescence microscope (Nikon, Tokyo, Japan) controlled with ZEN software (Carl Zeiss).

### Intracellular Free Ca^2+^ Detection

Free Ca^2+^ levels in the BMDMs were measured by staining with the Ca^2+^ -binding dye Fluo-3 AM. Briefly, the cells harvested after the specific treatments were washed twice with ice-cold PBS, and incubated with 5 μM Fluo-3 AM at 37°C for 30 min. The stained samples were washed three times with PBS and analyzed by flow cytometry on FACS Calibur (Beckman Coulter, United States).

### ASC Oligomerization

ASC oligomerization was determined as described previously with minor modifications (Lugrin and Martinon, 2017). Briefly, BMDMs or PMA-primed THP-1 cells were stimulated with LPS (500 ng/ml) for 6 h and then with ATP (5 mM) or nigericin (10 μM) for 1 h. The suitably treated cells were harvested by scraping in cold PBS containing 2 mM EDTA, and centrifuged at 1,500 × *g*. The cells were then lysed in Cold Buffer A (20 mM HEPES-KOH, pH 7.5, 10 mM KCl, 1.5 mM MgCl_2_, 1 mM EDTA, 1 mM EGTA, 320 mM sucrose) by repeatedly (20 times) passing through a 21-gauge needle, and the lysates were centrifuged at 3,300 × *g* to pellet the inflammasome complexes. The precipitate was resuspended in CHAPS buffer (20 mM HEPES-KOH, pH 7.5, 5 mM MgCl_2_, 0.5 mM EGTA, 0.1 mM PMSF, 0.1% CHAPS) containing 4 mM disuccinimidyl, and incubated for 30 min at room temperature with constant rotation to cross link the proteins. The samples were centrifuged at 5,000 × g for 10 min, and the pellets were harvested for western blotting.

### Histopathological Staining and Immunohistochemistry

The lung tissue paraffin sections were cleared in xylene and rehydrated through an alcohol gradient. After antigen retrieval, the slides were blocked using 20% normal goat serum and then incubated overnight with anti-CHOP and anti-GPR78 antibodies at 4°C. The slides were washed three times with PBS and incubated with HRP-linked secondary antibodies at room temperature. Diaminobenzidine-hydrogen peroxide (Sigma-Aldrich) was used to develop color and the nuclei were counterstained with 0.5% hematoxylin (Li et al., 2021).

### Statistical Analysis

The data from three independent experiments are presented as means ± SD. Statistical significance was determined using Student’s t-test and indicated as *p < 0.05 and **p < 0.01. All statistical analyses were performed using the GraphPad Prism software.

## Results

### 5-HMF Protects Mice Against Lung Injury and Inflammation Induced by Lipopolysaccharide

The chemical structure of 5-HMF is shown in Figure 1A. To evaluate the cytotoxicity of 5-HMF, we treated BMDMs and THP-1 cells with different concentrations of 5-HMF for 24 h and measured cell viability using MTS assay. As shown in Supplementary Figure S1, 5-HMF did not significantly affect the viability of BMDMs and THP-1 cells even at the high concentration of 100 μg/ml. To determine the therapeutic role of 5-HMF on LPS-induced pneumonia, we established a mouse model of ALI via intraperitoneal injection of LPS (10 mg/kg). H&E staining of the lung tissues indicated that compared to the untreated LPS-challenged mice, 5-HMF pre-treatment effectively reduced peri-bronchial wall thickening, infiltration of inflammatory cells into the alveolar space, and vascular congestion (Figure 1B). Furthermore, 5-HMF also attenuated the LPS-induced increase in the number of inflammatory cells such as macrophages, lymphocytes and neutrophils (Figure. 1C), as well as the total protein concentration and cell count (Figure 1D) in the BALF. MPO is released by activated neutrophils and is considered as a specific marker of neutrophil infiltration. As shown in Figure 1E, MPO activity in the lung tissues of the LPS-challenged mice was also reduced in 5-HMF treatment group. Consistent with this, the serum levels of proinflammatory cytokines including IL-6, TNF-α, IL-1β and mouse IL-8 homologue, keratinocyte-derived cytokine (KC) were significantly lower in the 5-HMF-treated versus untreated mice (Figures 1F–I). Unexpectedly, the serum level of anti-inflammatory cytokine IL-10 was repressed in 5-HMF treatment group (Figure 1J). To further assess the therapeutic effect of 5-HMF during acute inflammation, we monitored the survival of the LPS-challenged mice pre-treated with 5-HMF (6 or 12 mg/kg, i. p.) or PBS. As shown in Figure 1K, the overall survival of the mice pre-treated with 5-HMF was significantly prolonged over a 6-day period, and a greater protective effect was observed with the higher dose. Taken together, 5-HMF protected mice against LPS-induced lung inflammation and injury.

### 5-HMF Inhibits the Lipopolysaccharide-Induced Inflammatory Response in Mouse Macrophages and Human Monocytes

**FIGURE 2:**
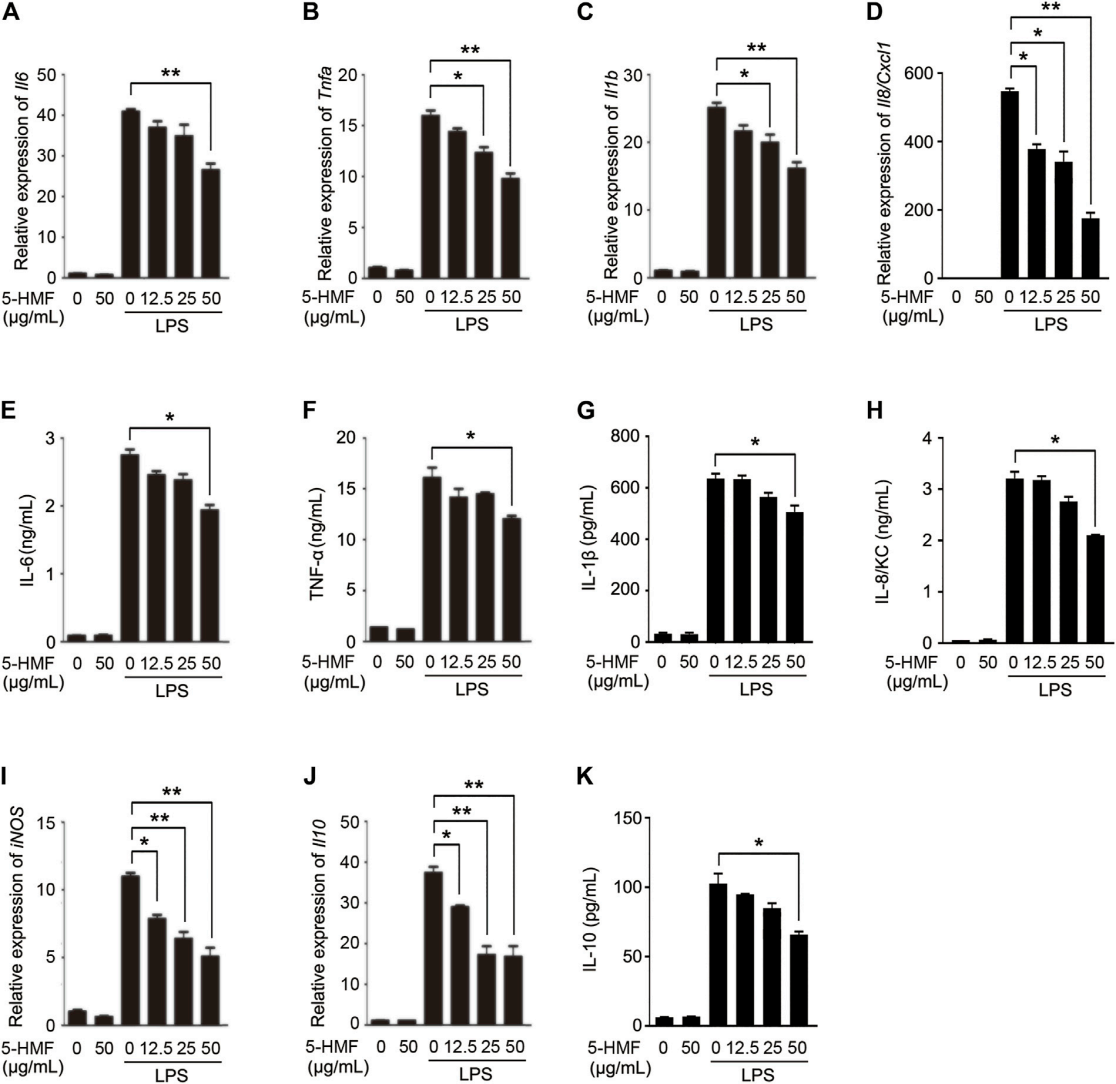
5-HMF inhibits the production of LPS-triggered proinflammatory cytokines in BMDMs. BMDMs were pretreated with PBS or 5-HMF and stimulated with LPS (100 ng/ml). **(A–D)** *Il6* **(A)** *, Tnfa* **(B)** *, Il1b* **(C)** *and Il8/Cxc1* **(D)** mRNA levels. **(E–H)** IL-6 **(E)**, TNF-α **(F)**, IL-1β **(G)** and IL-8/KC H) levels in the culture supernatant. **(I,J)** *iNOS* **(I)** and *Il10* **(J)** mRNA levels. **(K)** IL-10 levels in the culture supernatant. Data are the mean ± SD of three independent experiments: * and ** indicate *p* < 0.05 and *p* < 0.01 respectively by Student’s t-test.

Macrophages mediate both innate and adaptive immune response by producing inflammatory cytokines and presenting antigens, and also play a central role during lung inflammation and injury. To determine whether 5-HMF regulated the LPS-induced inflammatory response in macrophages, we treated murine BMDMs with different concentrations of 5-HMF (0–50 μg/ml) and stimulated them with LPS (100 ng/ml) for 6 h. As shown in Figures 2A–H, in BMDMs, 5-HMF treatment significantly decreased the LPS-induced production of IL-6, TNF-α, IL-1β and IL-8/KC, which was encoded by *Cxcl1* gene in mice (Figures 2A–H). Since nitric oxide (NO) is a critical mediator of ALI pathogenesis, we next analyzed the expression of inducible nitric oxide synthase (iNOS) and found that the *iNOS* mRNA levels were markedly lower in the 5-HMF-treated macrophages (Figure 2I). Unexpectedly, the mRNA level and the production of the anti-inflammatory cytokine IL-10 were also reduced following 5-HMF treatment (Figures 2J,K). Similar pharmacological effects of 5-HMF were observed in the THP-1 cells as well with a significant inhibition of LPS-induced IL-6, TNF-α, IL-1β, IL-8 (encoded by *CXCL8* gene in human), iNOS and IL-10 (Supplementary Figure S2). Taken together, 5-HMF suppressed the LPS-induced inflammatory response by reducing the production of inflammation-related chemokines and cytokines.

### 5-HMF Inhibits Lipopolysaccharide-Induced NF-κB Signaling Pathway

**FIGURE 3:**
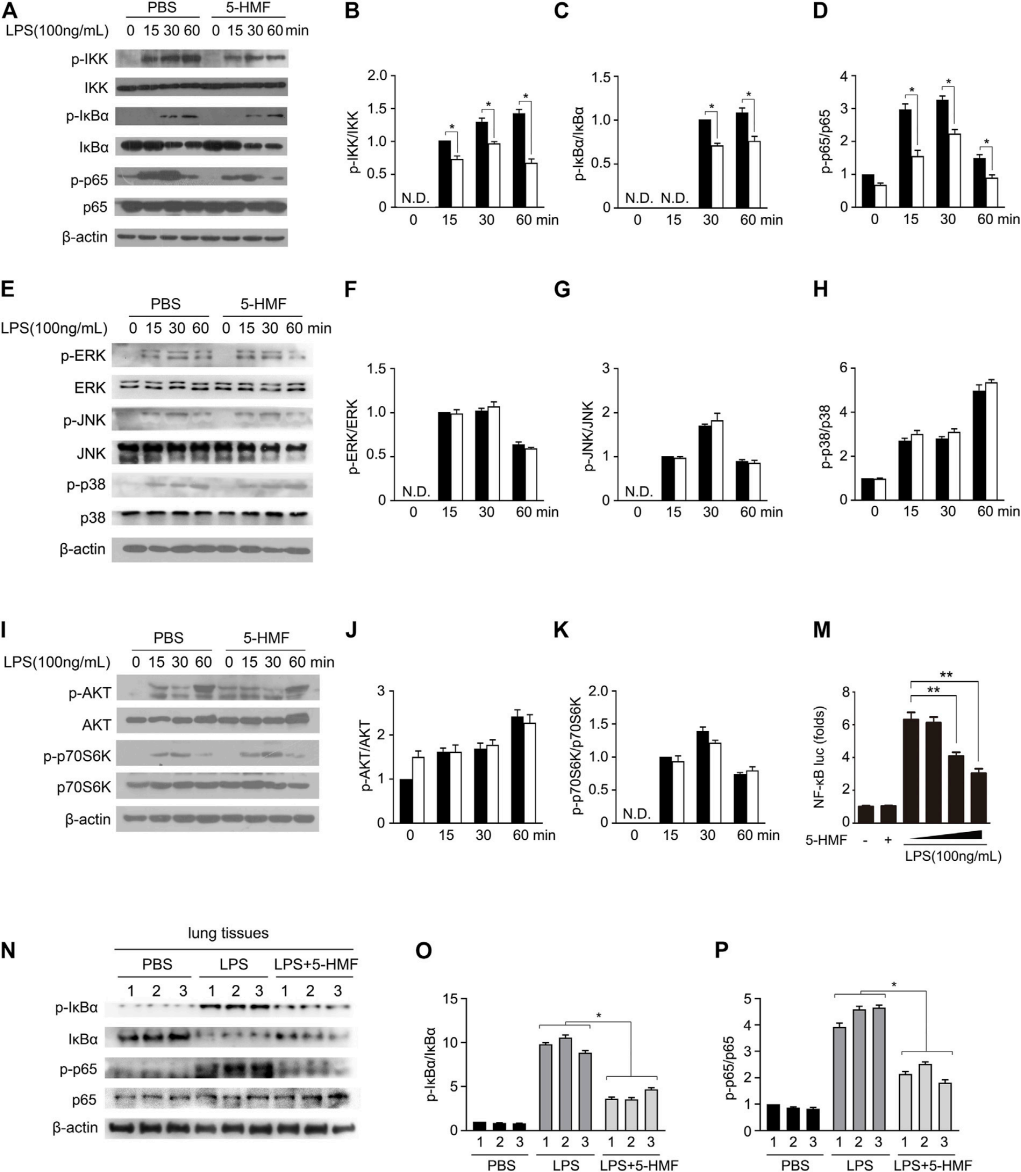
5-HMF inhibits NF-κB signaling pathway. **(A–K)** BMDMs pretreated with PBS or 5-HMF (50 μg/ml) were subsequently challenged by LPS for varying durations. **(A–D)** Immunoblot showing the expression levels of total and phosphorylated IKK, IκBα and p65 proteins **(A)**. **(B–D)** The relative band densities of the phosphorylation of IKK **(B)**, IκBα **(C)** and p65 **(D)** were measured by ImageJ software, N.D. means non-detected. **(E–H)** Immunoblot showing total and phosphorylated ERK, JNK and p38 proteins **(E)**. **(F–H)** The relative band densities of the phosphorylation of ERK **(F)**, JNK **(G)** and p38 **(H)**. **(I–K)** Immunoblot showing total and phosphorylated AKT and p70S6K proteins **(I)**. **(J, K)** The relative band densities of the phosphorylation of AKT **(J)** and p70S6K **(H)**. **(M)** RAW264.7 cells transfected with pGL3-NF-κB were pretreated with PBS or different concentrations of 5-HMF as indicated, and stimulated with LPS for 6 h. Luciferase activity of the NF-κB-driven promoter in the indicated groups. **(N–P)** Immunoblot showing total and phosphorylated IKK, IκBα and p65 in the lung tissues of mice treated as in Figure 1A N. **(O,P)** The relative band densities of the phosphorylation of IκBα **(O)** and p65 **(P)**. Data are the mean ± SD of three independent experiments: * and ** indicate *p* < 0.05 and *p* < 0.01 respectively by Student’s t-test.

Pro-inflammatory cytokines are produced following activation of NF-κB and MAPK signaling in response to TLR stimulation (Ko et al., 2020). To further elucidate the molecular mechanisms underlying the anti-inflammatory effects of 5-HMF, therefore, we analyzed the expression and phosphorylation levels of NF-κB and MAPK signal pathway mediators in the LPS-stimulated macrophages. The BMDMs were treated with 5-HMF, and thereafter with LPS for varying durations. 5-HMF treatment reduced the levels of phosphorylated IKKα/β, IκBα and p65 in the LPS-stimulated BMDMs (Figures 3A–D). We also performed a reporter gene assay by transfecting RAW264.7 cells with the NF-κB luciferase reporter plasmid, and found that 5-HMF repressed NF-κB-induced transcriptional activity in a dose-dependent manner (Figure 3M). However, 5-HMF did not affect the activation of the MAPK signaling molecules ERK, JNK and p38 (Figures 3E–H). The AKT/mTOR signaling pathway is also crucial to initiate and mediate the NF-κB signal transduction pathway and is associated with the inflammatory response (Meng et al., 2018). Therefore, we analyzed the levels of *p*-AKT, AKT, p-P70S6K and P70S6K in the suitably treated cells. As shown in Figures 3I–K, 5-HMF has no significant effect on the LPS-induced phosphorylation of AKT (Ser473) and p70S6K (Thr389). In the *in vivo* ALI model as well, 5-HMF pre-treatment significantly reduced phosphorylation levels of IκBα and p65 in the lung tissues following LPS-challenge compared to that in the untreated mice (Figures 3N–P). In conclusion, 5-HMF inhibited NF-κB activation in LPS-challenged macrophages, which might be the key mechanism underlying its anti-inflammatory effect against ALI.

### 5-HMF Inhibits NLRP3 Inflammasome Activation in Mouse Macrophages and Human Monocytes

**FIGURE 4:**
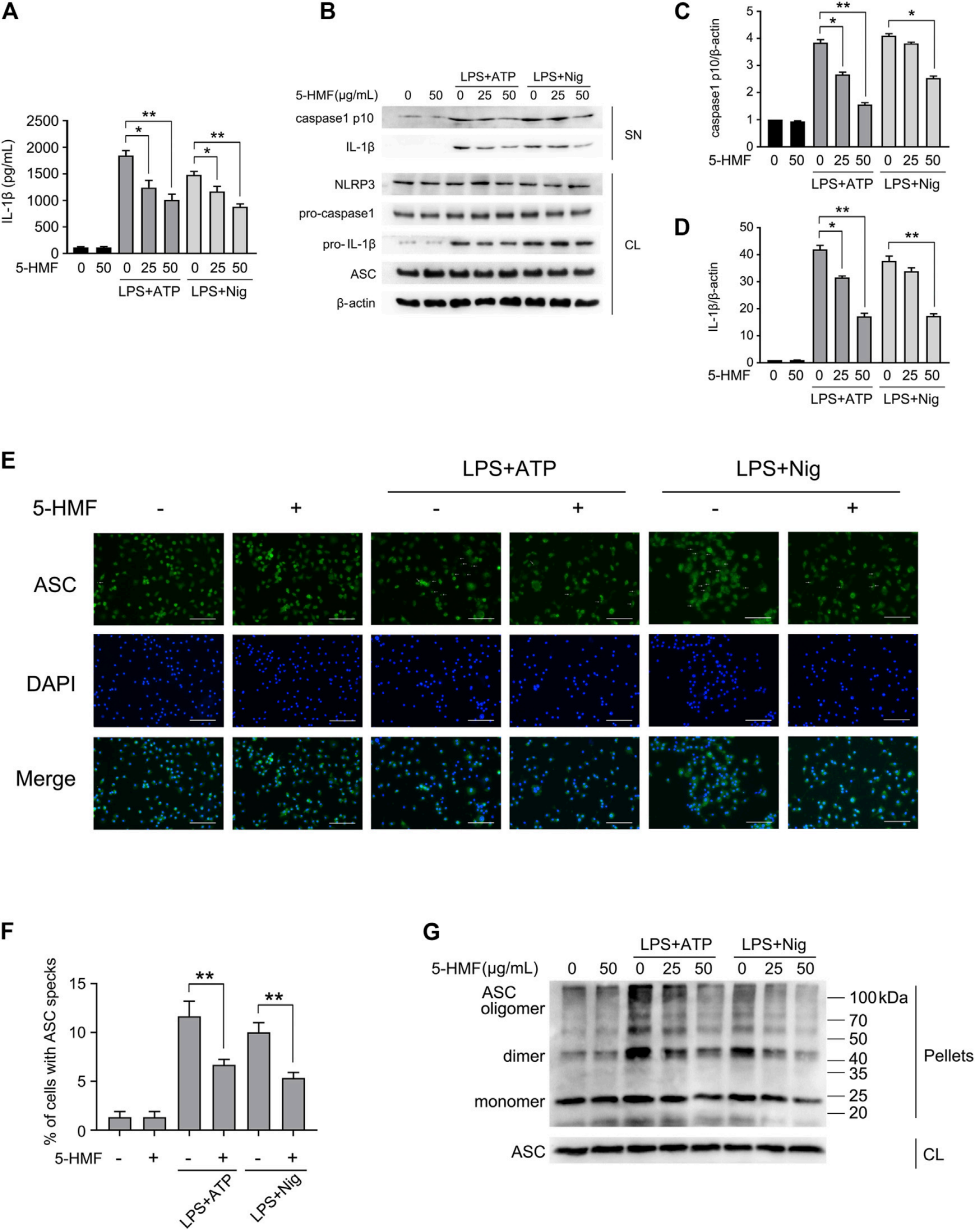
5-HMF suppresses NLRP3 inflammasome activation. BMDMs were pretreated with different concentrations of 5-HMF for 30 min as indicated, followed by LPS (500 ng/ml) for 6 h and ATP (5 mM) or nigericin (10 μM) for 1 h **(A)** IL-1β levels in the culture supernatants. **(B)** Immunoblot showing levels of indicated proteins in the culture supernatants (SN) and cell lysates (CL). **(C,D)** The relative band densities of IL-1β **(C)**, caspase-1 p10 **(D)** in the supernatant. **(E)** Representative immunofluorescence images showing ASC speck formation (×400), Scale bar, 500 μm. **(F)** The percentage of cells containing ASC-specks as in **(E)**. **(G)** Immunoblot showing ASC oligomerization in pellets cross-linked by DSS.

Inflammasomes are critical to the innate immune response and inflammation (Walsh et al., 2014). Herein, we determined the effects of 5-HMF on NLRP3 inflammasome activation in LPS-challenged BMDMs and THP-1 cells following stimulation with ATP or nigericin (Mariathasan et al., 2006). The BMDMs were sequentially exposed to different concentrations of 5-HMF, LPS and finally ATP or nigericin. 5-HMF inhibited the release of IL-1β from the BMDMs and THP-1 cells (Figure 4A, Supplementary Figure S4A), and also suppressed the maturation and secretion of cleaved caspase-1p10 and IL-1β in the LPS-primed BMDMs (Figures 4B–D) and THP-1 cells (Supplementary Figures S4B–D) following stimulation with ATP or nigericin. NLRP3 inflammasome assembly is initiated by the interaction of NLRP3 with ASC (Vajjhala et al., 2012; Cai et al., 2014; Lu et al., 2014). During this step, ASC monomers form large specks in cells via self-challenged oligomerization, which is a marker of NLRP3 inflammasome activation (Elliott and Sutterwala, 2015). Therefore, we surmised that 5-HMF may inhibit NLRP3 inflammasome activation by suppressing ASC oligomerization. We found that 5-HMF treatment significantly reduced the formation of ASC specks in the BMDMs (Figures 4E,F) and THP-1 cells (Supplementary Figures S4E, F) stimulated with LPS and ATP or nigericin. Furthermore, 5-HMF also decreased the levels of ASC dimers and oligomers in the DSS-crosslinked pellets of both BMDMs (Figure 4G) and THP-1 cells (Supplementary Figure S4G) stimulated as above. Taken together, 5-HMF blocks NLRP3 inflammasome activation in murine macrophages and human monocytes by repressing ASC oligomerization.

### 5-HMF Inhibits Lipopolysaccharide-Induced ER Stress

**FIGURE 5:**
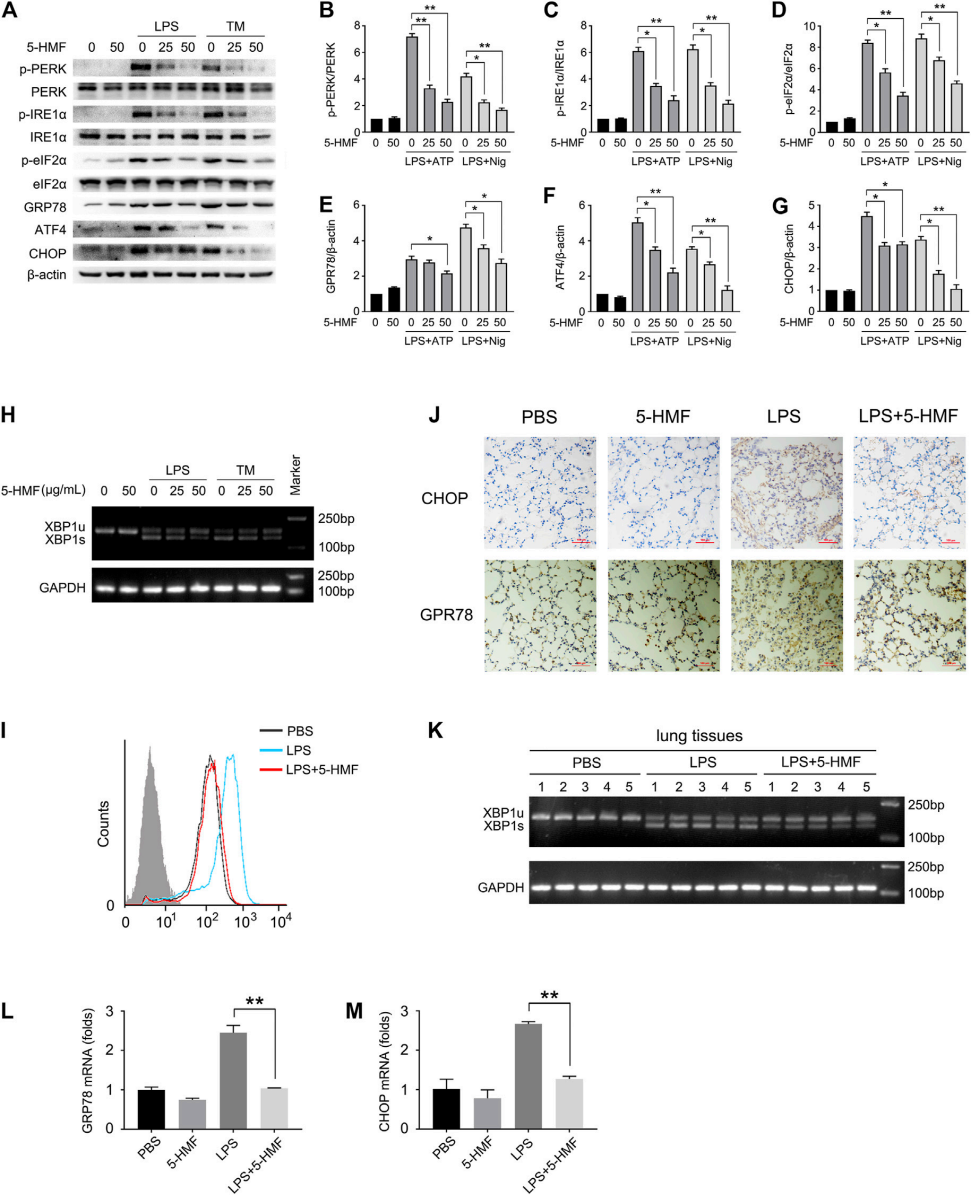
5-HMF inhibits LPS-triggered ER stress. **(A–K)** BMDMs pretreated with different concentrations of 5-HMF were challenged with LPS (500 ng/ml) for 6 h or tunicamycin (1 μg/ml) for 16 h **(A–G)** Immunoblot showing total and phosphorylated PERK, eIF2α, IRE-1α and expression levels of ATF4, GPR78 and CHOP in BMDMs **(A)**. **(B–G)** The relative band densities of the phosphorylation of PERK **(B)**, IRE1α **(C)** and eIF2α **(D)**, and the expression of GPR78 **(E)** ATF4 **(F)** and CHOP **(G)**. **(H)** RT-PCR analysis of XBP1 splicing in BMDMs cells treated with PBS or 5-HMF, followed by LPS or tunicamycin stimulation as indicated. **(I)** FACS analysis of the intracellular Ca^2+^ concentrations stained by green fluorescence (Fluo-3). **(J)** Representative IHC images showing *in-situ* expression of CHOP and GPR78 in lung tissues of mice treated as in Figure 1A (×200), Scale bar, 100 μm. **(K)** RT-PCR analysis of XBP1 splicing in lung tissues of mice treated with PBS or 5-HMF as in Figure 1A, followed by LPS stimulation. **(L,M)** *Chop* **(L)** and *Gpr78* **(M)** mRNA levels in lung tissues of mice treated as in Figure 1A. Data are the mean ± SD of three independent experiments: * and ** indicate *p* < 0.05 and *p* < 0.01 respectively by Student’s t-test.

Studies increasingly show that ER stress can affect NLRP3 inflammasome activation via the generation of reactive oxygen species (ROS), calcium or lipid metabolism, and the unfolded protein response (UPR) (Chen et al., 2019). To determine whether 5-HMF affected LPS-triggered ER stress in BMDMs, we additionally treated the cells with the ER stress inducer tunicamycin. As shown in Figures 5A–G, both LPS and tunicamycin individually increased the phosphorylation of PERK, IRE1α and eIF2α in the BMDMs, and upregulated GRP78, ATF4 and CHOP. These mediators of ER stress were markedly attenuated by 5-HMF in a dose-dependent manner (Figures 5A–G). Furthermore, 5-HMF also inhibited basal XBP-1 splicing induced by LPS or tunicamycin in BMDMs (Figure 5H). The differentially treated BMDMs were then stained with Fluo-3 to measure intracellular Ca^2+^ levels. As shown in Figure 5I, LPS significantly increased intracellular Ca^2+^ accumulation, which was remarkably downregulated in the presence of 5-HMF. In line with the *in vitro* findings, 5-HMF reduced XBP1 splicing in the lung tissues of LPS-challenged mice (Figure 5K), as well as the *in-situ* expression of CHOP and GPR78 proteins (Figure 5J) and mRNAs (Figures 5L,M). Altogether, our results suggested that 5-HMF inhibited LPS-induced ER stress both *in vitro* and *in vivo*.

### 5-HMF Inhibits NLRP3 Inflammasome Activation by Targeting Endoplasmic Reticulum Stress

**FIGURE 6:**
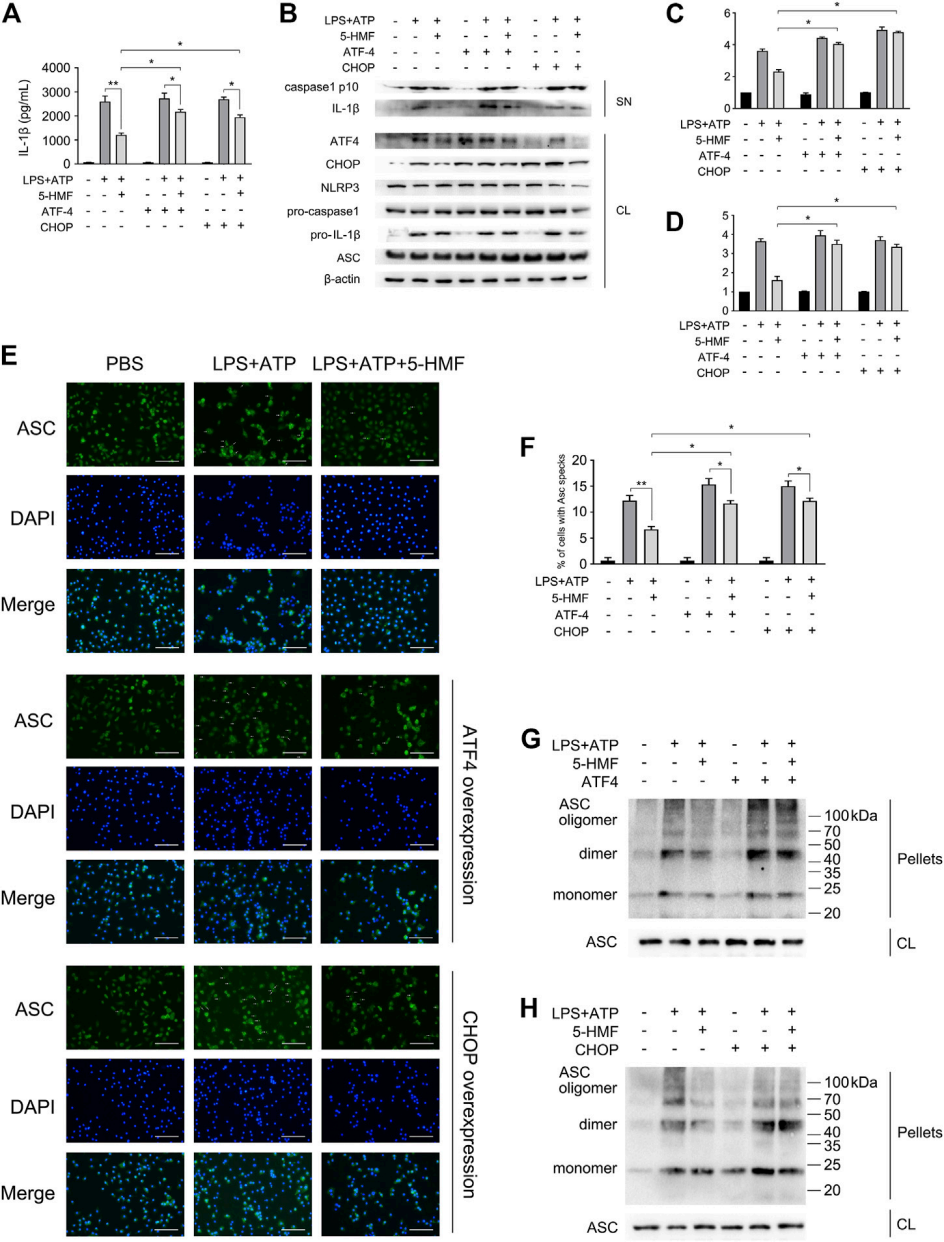
5-HMF inhibits NLRP3 inflammasome by down-regulating ER stress. BMDM were transfected with ATF4 and CHOP mRNA as indicated. After 48 h, cells were incubated with 5-HMF (50 μg/ml) or PBS for 30 min, followed by LPS (500 ng/ml) for 6 h and ATP (5 mM) for 1 h **(A)** IL-1β levels in culture supernatants. **(B)** Immunoblot showing levels of indicated proteins in the culture supernatants (SN) and cell lysates (CL). **(C,D)** The relative band densities of IL-1β **(C)**, caspase-1 p10 **(D)** in supernatant. **(E)** Representative immunofluorescence images showing ASC speck formation (×400), Scale bar, 500 μm. **(F)** The percentage of cells containing ASC-specks in **(E**). **(G,H)** Immunoblot showing ASC oligomerization in pellets cross-linked by DSS.

Given that ER stress activates NLRP3 inflammasome through various mediators (Compan et al., 2015), we next determined whether the inhibitory effect of 5-HMF on NLRP3 inflammasome activation involved the ER stress pathway. To this end, we overexpressed ATF4 or CHOP in the BMDMs by transfecting the respective mRNAs, and stimulated the cells with LPS plus ATP/Nigericin. As shown in Figure 6B, the protein levels of ATF4 or CHOP were upregulated after mRNA transfection, respectively. As expected, 5-HMF-driven negative regulation of ER stress-associated NLRP3 activation was reversed by ATF4 or CHOP over-expression (Figure 6). Furthermore, ATF4/CHOP overexpression also neutralized the inhibitory effect of 5-HMF on IL-1β release from the BMDMs following LPS plus ATP/nigericin stimulation (Figure 6A). Moreover, ATF4/CHOP overexpression partially abrogated the inhibitory effects of 5-HMF on the maturation and secretion of caspase-1 triggered by LPS plus ATP/nigericin stimulation (Figures 6B–D). Since 5-HMF suppressed ASC oligomerization, we also examined whether the attenuation of ER stress affected ASC speck formation and oligomerization. The results showed that overexpression of ATF4/CHOP restored ASC speck formation (Figures 6E,F) and ASC oligomerization (Figures 6G,H) in the cells stimulated with LPS and ATP even in the presence of 5-HMF. Taken together, attenuation of ER stress is crucial to the inhibitory effect of 5-HMF on NLRP3 inflammasome activation.

### Inhibition of Endoplasmic Reticulum Stress Is Essential for 5-HMF-Mediated Anti-Inflammatory Activity and Amelioration of ALI *In Vivo*

**FIGURE 7:**
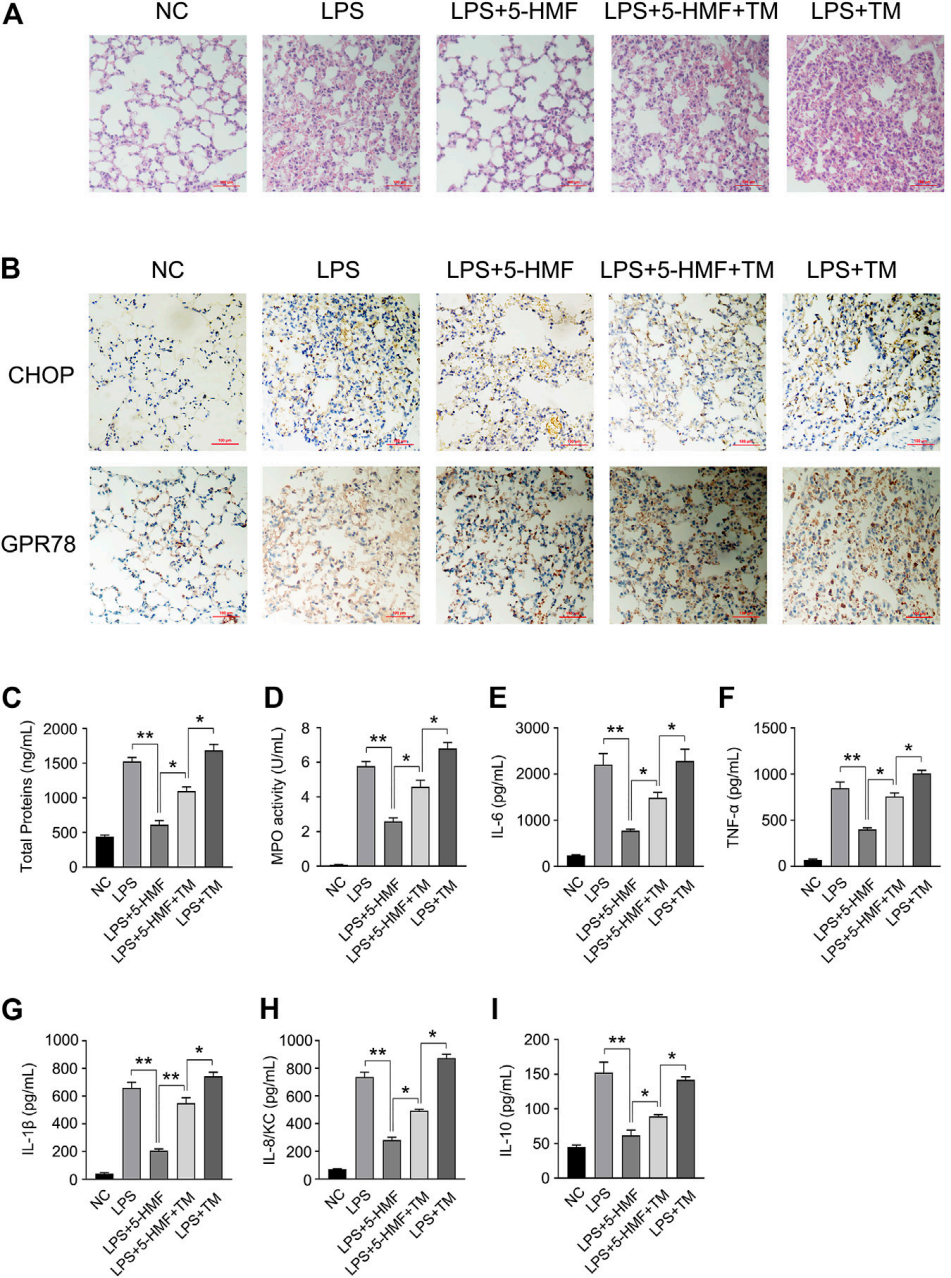
Inhibition of ER stress by 5-HMF mediates amelioration of LPS-induced acute lung injury. C57BL/6J mice (5 mice/group) pretreated with PBS or 5-HMF (12 mg/kg, i. p.) and/or tunicamycin (TM, 0.2 mg/kg, i. p.) were challenged with LPS (1 mg/kg, i. p.). **(A)** Representative images of H&E-stained lung tissue sections from the indicated groups (×200), Scale bar, 100 μm. **(B)** Representative IHC images showing *in-situ* expression of CHOP and GPR78 in lung tissues of mice (×200), Scale bar, 10 μm. **(C)** Protein content in BALF. **(D)** MPO activity of lung tissues. **(E–I)** IL-6 **(E)**, TNF-α (F), IL-1β **(G)**, IL-8/KC **(H)** and IL-10 **(I)** levels in sera. All results are expressed as the mean ± SD: * and ** indicate *p* < 0.05 and *p* < 0.01 respectively by Student’s t-test.

To further explore the role of ER stress to the action mode of 5-HMF in the amelioration of pulmonary dysfunction in the mice model of ALI, we took advantage of tunicamycin (TM), a well-known ER stress inducer in the LPS-triggered ALI mice model to confirm the action of 5-HMF. As shown in Figure 7, tunicamycin significantly reversed the protective effects of 5-HMF on LPS-induced inflammation and lung injury. Mice co-treated with tunicamycin displayed more severe signs of LPS-induced lung inflammation and injury compared to the non-treated controls (Figure 7A). In addition, the downregulation of CHOP and GPR78 by 5-HMF were reversed by tunicamycin treatment (Figure 7B). The total protein level in BALF (Figure 7C), MPO activity (Figure 7D), and the levels of IL-6, TNF-α, IL-1β, IL-8/KC and IL-10 in the LPS-challenged mice were exacerbated by tunicamycin pre-treatment compared to mice that received only LPS and 5-HMF (Figures 7E–I). To summarize these results, attenuation of ER stress contributed substantially to the inhibition of the NLRP3 inflammasome in the ALI model treated with 5-HMF. Thus, 5-HMF protected against NLRP3 inflammasome-mediated pulmonary dysfunction by modulating ER stress.

## Discussion

ALI is a severe lung disease characterized by pulmonary and capillary edema, which eventually leads to acute respiratory failure (Ware and Matthay, 2000). Excessive pulmonary infiltration of inflammatory cells and overproduction of proinflammatory cytokines are the key pathological factors of ALI (Belchamber and Donnelly, 2017). Thus, it is crucial to identify novel anti-inflammatory compounds for the prevention and treatment of inflammation-relevant lung injury. 5-HMF is an active compound commonly found in sugar-rich heat-processed foods, including honey, coffee, dried fruit and fruit juices (Shapla et al., 2018; Zou et al., 2021). Studies show that 5-HMF exerts anti-inflammatory or anti-allergic effects (Zhao et al., 2013; Alizadeh et al., 2017), and can inhibit ROS production and the expression of inflammatory factors such as PGE2, IL-1β, IL-6 and TNF-α in LPS-stimulated RAW264.7 macrophages (Zhao et al., 2013). In this study, we investigated the effect of 5-HMF on ALI and explored the underlying molecular mechanisms. Our findings indicated that 5-HMF protects against LPS-induced ALI by inhibiting the pro-inflammatory NF-κB signaling cascade and the activation of NLRP3 inflammasome, which involves attenuation of the ER stress pathway.

The potential mechanisms of ALI have been explored in LPS-stimulated mouse models (Liu et al., 2018). LPS stimulation led to significant histological changes such as vascular leakage, neutrophils infiltration, enhanced MPO levels and activity in the BALF, and over-production of various inflammatory cytokines (IL-6, TNF-α and IL-1β). In our study, we have reported for the first time that exogenous 5-HMF can ameliorate LPS-induced ALI, as indicated by the lower degree of pathological damage (Figure 1B) and reduced serum levels of inflammatory cytokines (Figures 1C–J). In addition, the survival rate of the LPS-challenged mice was markedly improved after 5-HMF treatment (Figure 1K). Activated macrophages play a pivotal role in the early stages of LPS-induced inflammation in the lungs by secreting large amounts of proinflammatory cytokines such as IL-6 and TNF-α, which in turn activate a complex signaling network that culminates in ALI (Lv et al., 2016). Likewise, human macrophages or monocytes cultured *in vitro* in the presence of LPS also produce significantly higher levels of proinflammatory cytokines (Wang et al., 2019). We found that 5-HMF neutralized LPS-induced production of IL-6, TNF-α and IL-1β by primary BMDMs or THP-1 monocytes in a dose-dependent manner, and even modulated the mRNA expression of iNOS and the anti-inflammatory cytokine IL-10 (Figure 2 and Supplementary Figure S2). These results indicated that 5-HMF protects against LPS-induced ALI by reducing the production of proinflammatory cytokines.

To further explore the mechanism underlying the action of 5-HMF, we analyzed its effects on the NF-κB signaling pathway and NLRP3 inflammasome activation. The NF-κB pathway plays a crucial role in inflammatory immune responses and is activated in various inflammatory diseases. Inactivation of NF-κB directly leads to decreased production of IL-6, TNF-α, IL-1β and other proinflammatory factors, which is capable of relieving LPS-induced ALI (Dang et al., 2016). Several natural compounds present in edible plants and TCM formulations exert anti-inflammatory effects by inhibiting the NF-κB signal pathway (Seo et al., 2018; Sahukari et al., 2020). Kong et al. have reported that 5-HMF inhibited this pathway in RAW264.7 cells. Consistent with this, we found that 5-HMF inactivated the NF-κB pathway in primary BMDMs and THP-1 monocytes exposed to LPS (Figures 3A–D and Supplementary Figure S3), which was confirmed by the luciferase reporter gene assay (Figure 3M). In the *in vivo* model as well, 5-HMF downregulated the NF-κB pathway in the lung tissues of LPS-treated mice (Figures 3N–P). The MAPK and AKT signaling pathways are also involved in the production of inflammatory and anti-inflammatory cytokines (Choi et al., 2014; Luo et al., 2019). However, 5-HMF had no significant effect on either MAPK or AKT signaling pathway in LPS-primed BMDMs even at a high concentration of 50 μg/ml (Figures 3E–K). This contradicts a previous report of the modulatory effect of 5-HMF on LPS-induced MAPK and AKT signaling pathway in RAW264.7 cells (Kong et al., 2019). This discrepancy could be due to different cellular models and/or different concentrations of 5-HMF. Nevertheless, our findings indicate that 5-HMF mitigates the inflammatory immune response by targeting the NF-κB signaling pathway.

The activation of NLRP3 inflammasome is responsible for the release of proinflammatory cytokines such as IL-1β and IL-18. The NLRP3 inflammasome is activated during the process of lung injury in response to cellular stress (Lee et al., 2016). Studies show that inactivation of the NLRP3 inflammasome due to chemical compounds or genetic mutations can markedly suppress ALI and other inflammatory diseases in animal models (Youm et al., 2015; Coll et al., 2015). However, no study so far has elucidated the regulatory effect of 5-HMF on NLRP3 inflammasome activation. The activation of NLRP3 inflammasome is initiated by the upregulation of NLRP3 and pro-IL-1β by LPS or other TLR agonists, and is followed by the assembly of NLRP3, caspase-1 and ASC, which was activated in response to various of stimuli, including ATP, nigericin or MSU crystals (Guo et al., 2015). We found that while 5-HMF inhibited the NF-κB pathway during LPS stimulation, it did not affect NLRP3 or pro-caspase-1 expression in the LPS-primed BMDMs and THP-1 cells. In addition, the expression of pro-IL-1β was only slightly attenuated by 5-HMF treatment (Figure 4B, Supplementary Figure S4B). However, 5-HMF did attenuate the maturation and secretion of IL-1β and caspase-1 in the LPS-primed primary BMDMs and THP-1 cells stimulated by ATP or nigericin (Figures 4A–D and Supplementary Figures S4A–D). Furthermore, 5-HMF also blocked ASC speck formation and ASC oligomerization, which precede NLRP3-caspase 1-ASC-complex formation and activation of NLRP3 inflammasome (Figures 4E–G and Supplementary Figures S4E–G) (Lu et al., 2014). Taken together, 5-HMF may alleviate LPS-induced inflammation and ALI by inactivating the NLRP3 inflammasome.

The ER stress response is closely related to inflammatory diseases such as liver injury, atherosclerosis, diabetes and ALI (Mohan et al., 2019; Lebeaupin et al., 2018). Studies show that the ER stress pathway is involved in the pathological process of lung injury (Endo et al., 2005; Kim et al., 2015b; Leonard et al., 2019). LPS challenge can significantly elevate the expression and/or activation of ER stress mediators including GRP78 protein (Aggarwal et al., 2018), PERK/eIF2-α/ATF4/CHOP pathway (Huang et al., 2020) and the activated splicing of XBP-1 (Zhao et al., 2020). A previous study on LO2 hepatocytes showed that 5-HMF can effectively attenuate ER stress induced by GalN/TNF-α and protect cells against apoptosis (Jiang et al., 2015). To determine whether the protective effect of 5-HMF on LPS-induced ALI depends on the attenuation of ER stress, we analyzed the expression and activation of crucial ER stress mediators. LPS or tunicamycin treatment significantly increased the phosphorylation of PERK, IRE1α and eIF2α, as well as the expression of GRP78, ATF4 and CHOP, all of which were inhibited by 5-HMF (Figures 5A–G). Consistent with this, we also showed that 5-HMF administration suppressed the significantly increased expression of CHOP and GRP78 induced by LPS in the lung tissues of ALI model mice (Figures 5J,L,M). In addition, 5-HMF significantly reduced LPS-induced XBP-1 mRNA splicing in BMDMs and in the murine lung tissues (Figures 5H,K). Since ER acts as the most important reservoir of calcium, Ca^2+^ homeostasis is disturbed during ER stress (Krebs et al., 2015; Stewart et al., 2015), resulting in mitochondrial depolarization, overproduction of ROS and induction of inflammatory responses (Rizzuto et al., 2012; Lebeaupin et al., 2018). Thus, treatments that inhibit calcium release from the ER may protect against cell injury and death. We found that 5-HMF suppressed LPS-induced calcium elevation in the cytoplasm of BMDMs (Figure 5I), which is consistent with its anti-inflammatory action. Taken together, 5-HMF protects against LPS-induced ALI and alleviates inflammatory responses by alleviating ER stress.

ER stress-mediated UPR is also a key factor involved in the activation of NLRP3 inflammasome, and the subsequent maturation and secretion of IL-1β (Menu et al., 2012). CHOP is a critical transcriptional factor during the progress of ER stress signaling, and its expression is triggered by three distinct stress signaling cascades. PERK induces CHOP expression by upregulating ATF4 (Ma et al., 2002), IRE1α triggers XBP1 mRNA splicing, and the mature XBP1s protein enhances CHOP expression by activating JNK (Dai et al., 2014). In addition, ATF6 directly upregulates CHOP (Yoshida et al., 2000). The overexpression of CHOP induces NLRP3 inflammasome activation, resulting in the maturation and release of IL-1β, caspase-1 and caspase-11, which eventually leads to pyroptotic cell death (Lebeaupin et al., 2015). Since 5-HMF abrogated LPS-induced ER stress by downregulating ATF4 and CHOP, we overexpressed ATF4 and CHOP mRNA in the BMDMs, and found that increased levels of IL-1β and caspase 1 were released following LPS and ATP stimulation (Figures 6A–D). This is consistent with the fact that the inhibitory activity of 5-HMF on NLRP3 inflammasome is partially dependent on the down-regulation of PERK/ATF4/CHOP signaling pathway. In addition, the suppression of ASC speck formation and ASC oligomerization by 5-HMF were also rescued upon ectopic expression of ATF4 or CHOP (Figures 6E–H). Thus, 5-HMF prevented NLRP3 activation *in vitro* via suppression of ER stress. In addition, we also demonstrated that 5-HMF-driven suppression of ER stress ameliorated lung injury and decreased inflammatory responses in LPS-induced ALI mice. Consistent with this, the ER stress inducer tunicamycin overcame the ameliorative effects of 5-HMF on LPS-induced pulmonary dysfunction. Tunicamycin also reversed the anti-inflammatory effect of 5-HMF, which mitigated the infiltration of inflammatory cells into the pulmonary alveoli, the MPO activities in BALF and the levels of proinflammatory cytokines in sera (Figure 7). Taken together with the above *in vivo* results, 5-HMF-driven ER stress inhibition is a major mechanism underlying the inactivation of NLRP3 inflammasome and amelioration of pulmonary dysfunction in ALI.

To summarize, our present study demonstrates a novel therapeutic effect of 5-HMF against LPS-induced ALI. 5-HMF not only inhibits the NF-κB-dependent inflammatory pathway but also inactivates the NLRP3 inflammasome by attenuating ER stress. Thus, 5-HMF should be studied further as a novel anti-inflammation drug. Given that 5-HMF is present in various natural foods and TCM formulations, our study provides a new pharmacological approach for lessening inflammation by using natural occurring compounds.

## Data Availability

The original contributions presented in the study are included in the article/Supplementary Material, further inquiries can be directed to the corresponding authors.
