# Supplementary figures and images for: 5-Hydroxymethylfurfural Alleviates Inflammatory Lung Injury by Inhibiting Endoplasmic Reticulum Stress and NLRP3 Inflammasome Activation
Source: Front Cell Dev Biol. 2021 Dec 13;9:782427. doi: 10.3389/fcell.2021.782427 (PMC8711100; doi:10.3389/fcell.2021.782427)

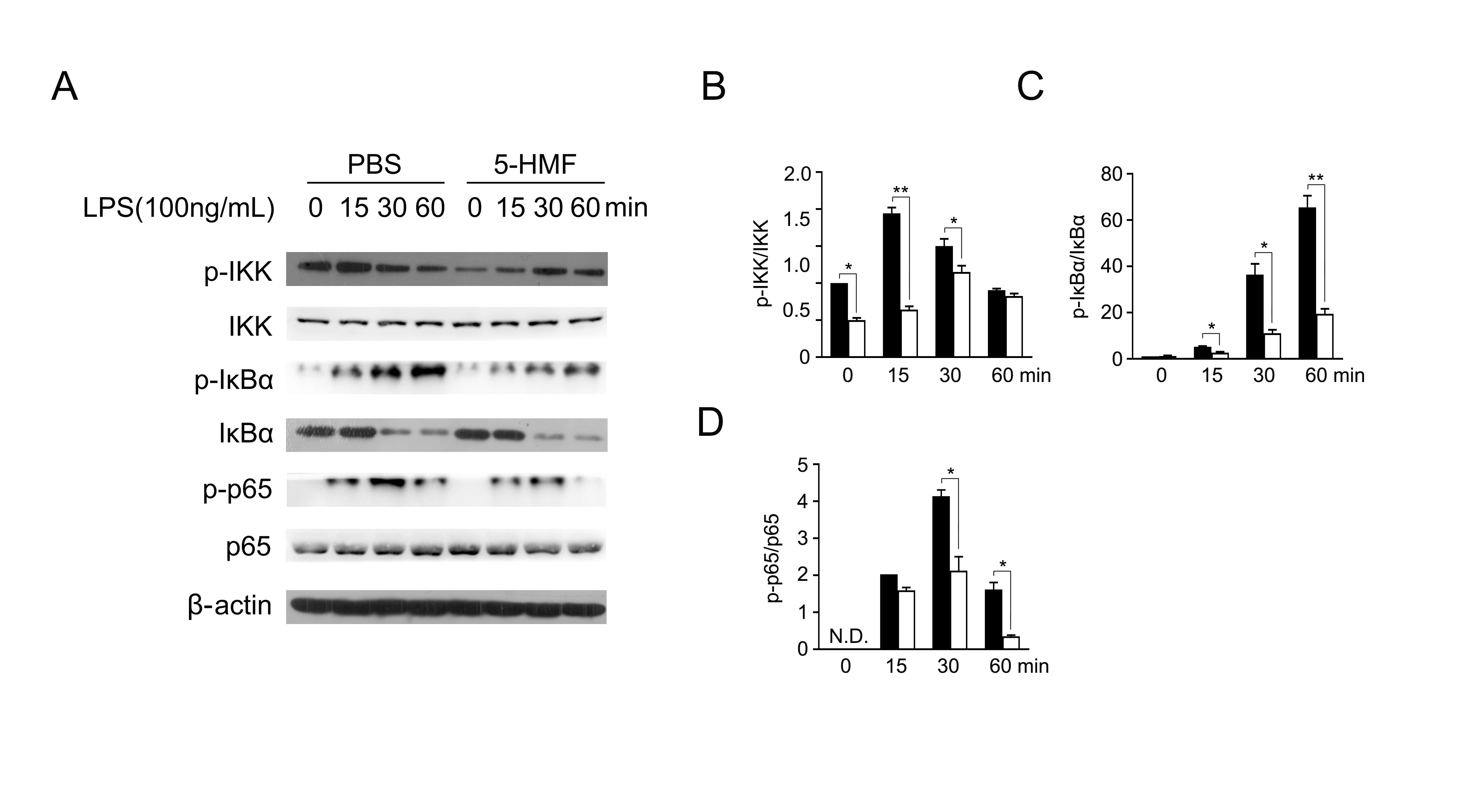

Supplement: Supplementary file 1 [file Image3.TIF]

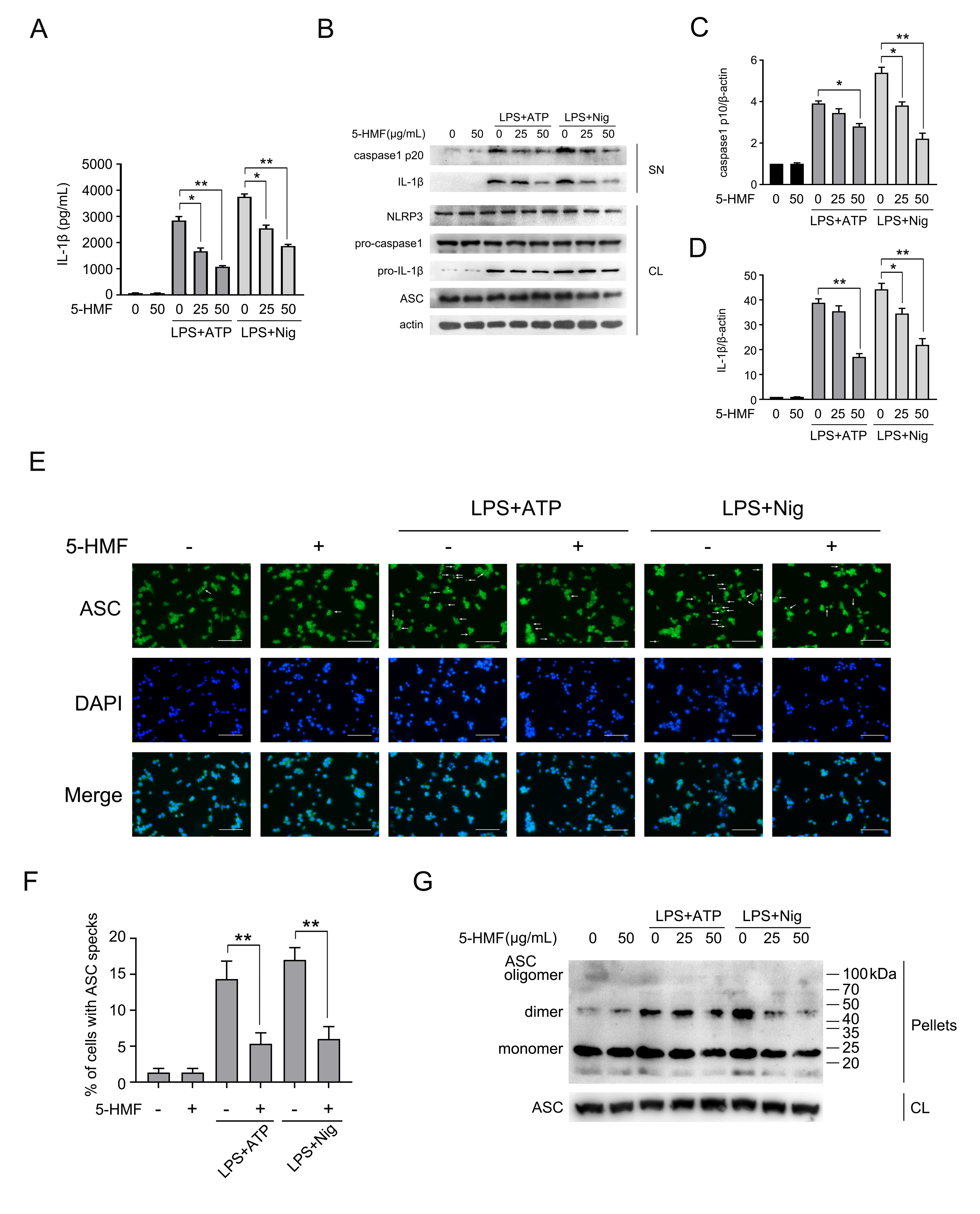

Supplement: Supplementary file 2 [file Image4.TIF]

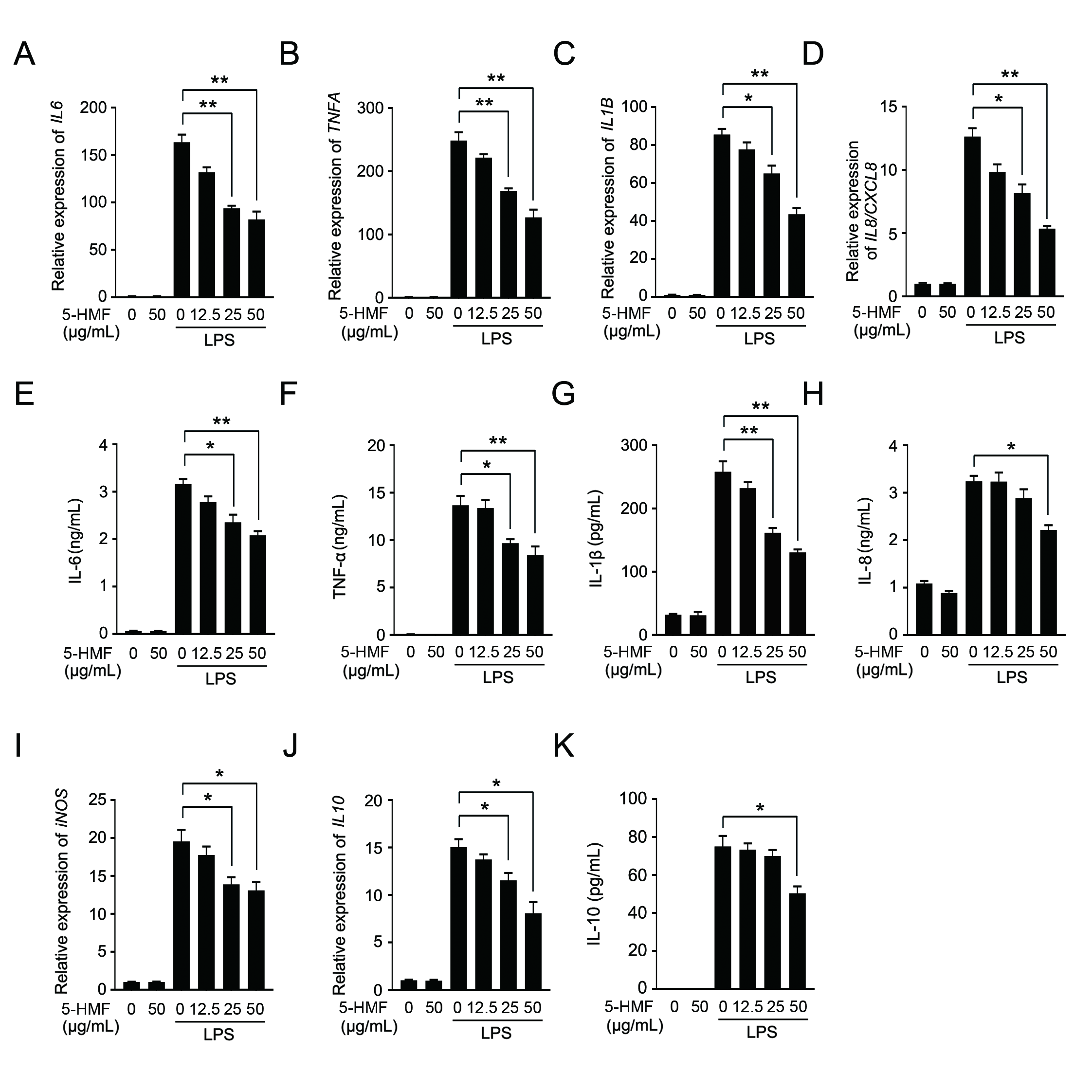

Supplement: Supplementary file 3 [file Image2.TIF]

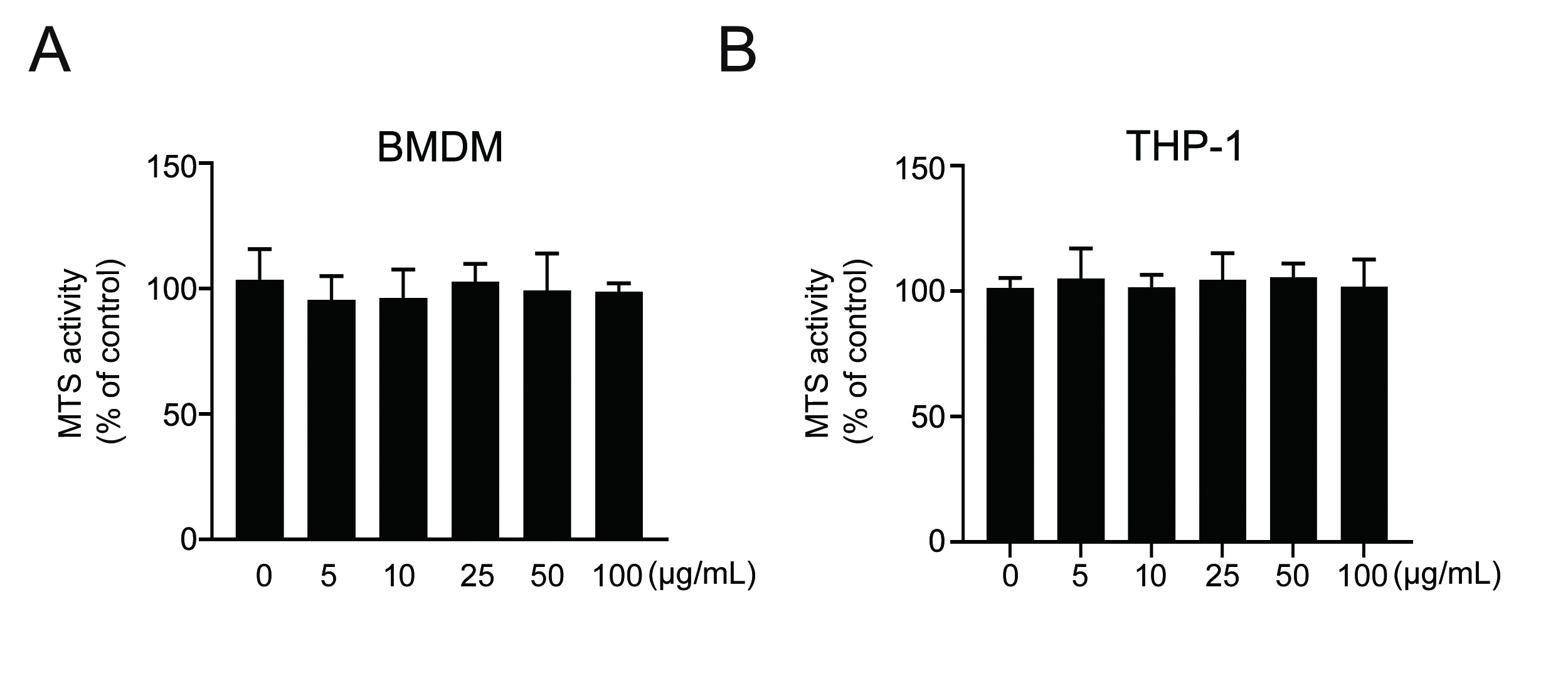

Supplement: Supplementary file 4 [file Image1.TIF]

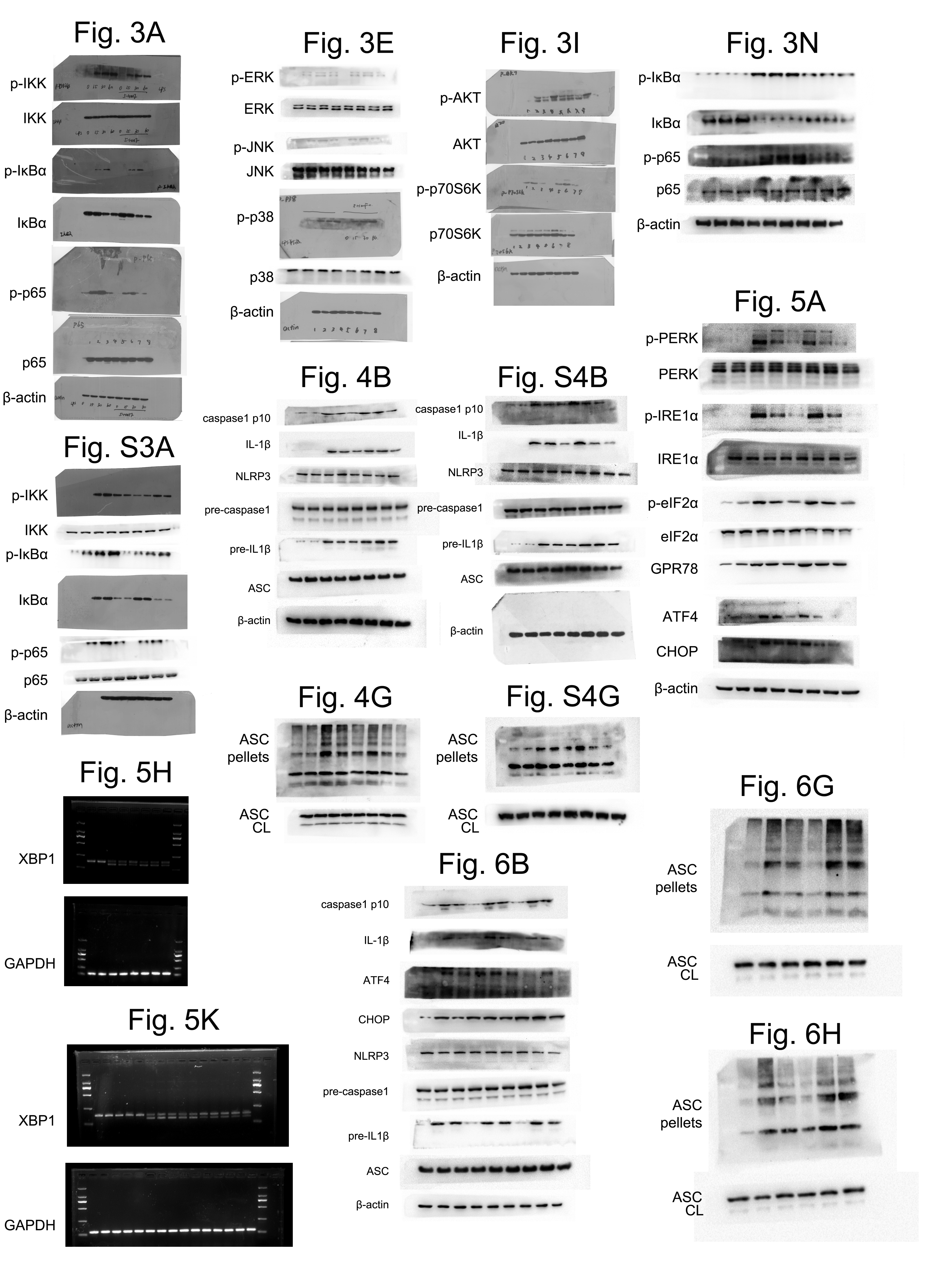

Supplement: Supplementary file 5 [file Image5.TIF]
